# Impact of prior exposures on biomarkers of blast during military tactical training

**DOI:** 10.3389/fneur.2025.1589742

**Published:** 2025-05-13

**Authors:** Zhaoyu Wang, Shengnan Sun, Qingkun Liu, Alis Askar Kranfli, Jeffrey Nemes, Molly Sullan, Andrew Hoisington, Lisa A. Brenner, Maciej Skotak, Christina R. LaValle, Yongchao Ge, Walter Carr, Fatemeh Haghighi

**Affiliations:** ^1^James J. Peters VA Medical Center, Bronx, NY, United States; ^2^Icahn School of Medicine at Mount Sinai, New York, NY, United States; ^3^Walter Reed Army Institute of Research, Silver Spring, MD, United States; ^4^Department of Physical Medicine and Rehabilitation, University of Colorado – Anschutz Medical Campus, Aurora, CO, United States; ^5^Rocky Mountain Regional VA Medical Center, Aurora, CO, United States; ^6^Air Force Institute of Technology, Wright-Patterson Airforce Base, OH, United States

**Keywords:** blast overpressure, mTBI, gene expression, biomarker, breacher, amyloid beta

## Abstract

**Introduction:**

Blast injuries and subclinical effects are of significant concern among those Service Members (SMs) participating in military operations and tactical trainings. Studies of SMs repeatedly exposed during training find concussion-like symptomology with transient decrements in neurocognitive performance, and alterations in blood biomarkers. How prior mild TBI (mTBI) history interacts with low-level blast (LLB) exposure, however, remains unexplored, which we investigate in the present study, to identify interindividual biomarker changes from LLB exposures influenced by prior history of mTBI.

**Methods:**

Gene transcript and amyloid-beta (Aβ40 and Aβ42) protein levels were assayed using timeseries blood specimens collected at pre-blast, post-blast (within ~1 h), and follow-up-blast (~16 h) after LLB exposure for 30 SMs (age 30.3 ± 7.5) via RNA-seq and Single Molecule Array (SIMOA). Statistical models with timepoint and mTBI status interaction adjusted for age were used, and *p*-values adjusted for multiple testing.

**Results:**

We found enrichment of genes involved in blood brain barrier, inflammatory, and immune responses associated with blast exposure, with significant elevated expression of target genes among SMs with mTBI history. Levels of Aβ40 and Aβ42 did not differ pre-blast vs. post/follow-up-blast LLB exposure when comparing SMs by prior mTBI history. Aβ40 and Aβ42 levels were significantly decreased in response to blast at the follow-up (~16 h) LLB exposure timepoint, concomitant with elevated expression of genes involved in amyloid-beta regulation and clearance in SMs with mTBI.

**Conclusion:**

Findings show inter-individual differences in biomarker levels following exposures to blast that may be attributed to prior mTBI history.

## Introduction

Injuries from exposure to explosive blasts rose dramatically during Operation Iraqi Freedom and Operation Enduring Freedom (OIF/OEF) due to the increased use of improvised explosive devices (IEDs) in military settings. It has been estimated that 10–20% of returning OIF/OEF Veterans have sustained a traumatic brain injury (TBI) (1), with many of these Veterans having symptoms suggestive of the residual effects of mild TBIs (mTBIs; persistent post concussive symptoms [PPCS]) that may not have been recognized prior to separation from service (2). Blast injuries and blast-related mTBIs are marked by their lack of immediate physiological or neurological symptoms, as well as a lack of detectable lesions in traditional neuroimaging screens (3). Given the lack of persistent symptomology from sub-concussive events available for diagnoses and proper treatment modalities at condition onset, sub-concussive injuries typically remain undiagnosed. Exposure to blast is associated with various disruptions in brain connectivity and deficits in cognitive functioning, including reduced white matter microstructural integrity (4), and decreases in neuronal activity (5), as well as cortical thinning in frontal lobe regions and worse symptom reporting associated with reductions in executive functioning and cognitive performance (6, 7). Undetected, these effects from blast can lead to the potential for symptoms to worsen over time, wherein undiagnosed sub-concussive injuries can result in short-term problems with operational readiness, and evolving conditions in the long-term including cognitive impairment, post-traumatic stress disorder (PTSD), depression, and suicidal behavior.

In the past decade, neuroimaging studies have revealed structural and functional brain changes associated with blast exposures (4, 8–24) that are supported by findings with blood biomarkers that have begun to provide insight into the underlying pathophysiological mechanisms of blast-related TBI. Notably the mechanisms and dynamic course of neuronal, axonal, and astroglial damage, supported by findings of increased blood serum levels of associated protein biomarkers such as Ubiquitin carboxy-terminal hydrolase L1 (UCHL1), neurofilament light chain (NfL) and total tau, and S100 calcium-binding protein B (S100-B) and Glial fibrillary acidic protein (GFAP) (25–28). Investigations of these biomarkers in context of blast exposures indicate that many, such as UCHL1 and GFAP, peak sharply in the hours following blast exposure, while others, including NfL and total tau, accumulate gradually and remain elevated for weeks following injury (29–32). Other CNS-based biomarkers linked to repetitive blast exposure include amyloid-beta (Aβ) proteins implicated in the etiology of neurodegenerative diseases such as Alzheimer’s disease. Findings from both animal (33) and human (34) studies indicate increased clearance and lower circulating levels of Aβ-40, −42, and precursor proteins (35) in the days initially following blast exposure. Whereas long-term, blast exposure has been associated with an increase in the accumulation of these same proteins in both brain (36) and peripheral blood (37). Exposure to blast has also been found to result in increased levels of inflammatory biomarkers acutely following exposure (32, 38) as well as chronically in the absence of acute exposures in individuals with long-term histories of blast (29, 39, 40). Additionally, longitudinal studies using timeseries data collected during military tactical training operations have shown that blast exposure is capable of inducing transcriptional and regulatory changes in military breachers (32, 35, 41, 42).

Despite such findings showing biomarker alterations in those with acute and chronic blast exposures, lacking are studies that explore the relationship between them by investigating inter-individual differences in biomarker response to blast exposures that may be driven by the individual’s prior lifetime exposures, such as prior history of TBI. In fact, studies of sports injuries have shown that history and frequency of sub-concussive injuries are associated with elevation of neurotrauma and inflammatory biomarkers (43–45). Blood biomarker data from these studies differentiate athletes with prior histories of sub-concussive injury from control athletes acutely post-injury, revealing that athletes with prior histories of sub-concussive injury exhibit greater levels of NfL, S100β (43), MCP-1 and MCP-4 (45). These findings underscore the need to also better understand and characterize changes in blast associated biomarker response that may be impacted by the individuals’ TBI history. In the present study we set out to investigate whether changes in gene transcripts and CNS-related biomarkers associated with blast exposures during tactical training are influenced by prior exposure load (i.e., prior history of mTBI). We hypothesize that prior history of mTBI will impact responsivity to blast as measured by differential transcriptional regulatory and CNS-related blood biomarker responses pre-versus post-blast exposures using serially collected blood samples during training.

## Methods

***Human Subjects***: All subjects consented to participate in the study and the human use protocol for interaction with the subjects was approved by the Institutional Review Board (IRB) of the Walter Reed Army Institute of Research (WRAIR, Silver Spring, MD; FYSA: WRAIR #2304) and chains of command prior to data collection. The procedures were followed in accordance with the ethical standards of the IRB, Army Regulation 70–25, and the Helsinki Declaration.

***Exposure and sample characteristics***: Data were collected at three timepoints from a single military training site, where 30 male subjects participated in a routine training course using explosives in the demolition of a concrete wall. As an observational study, the field team followed guidelines of minimal interference with daily activities of the hosting unit. We followed the pre-approved timeline which was consulted with and approved by the command of the host unit. The training took place at a military site as part of military breaching course. The details of the heavy wall breaching were previously reported (46, 47). Briefly, the subjects wear standard personal protective equipment (PPE) for this type of training: (1) helmet, (2) body armor, (3) eye protection, and (4) double hearing protection. The subjects were arranged in a “stack,” a line of 5–7 subjects hiding behind a ballistic blanket and positioned at 45° from the charge. The breaching exercises were conducted on walls constructed on-site using reinforced concrete-filled cinderblock or chain link fence using 10.0 and 15.0 lb. TNT equivalent charges, respectively. The overpressure associated with the charge detonations was captured using BlackBox Biometrics (B3) Blast Gauge® wearable sensors mounted on the shoulders and helmet. The average peak overpressure and impulse values were 5.0 ± 1.4 psi and 11.3 ± 1.3 psi·ms (concrete), and 6.2 ± 1.5 psi and 12.4 ± 1.4 psi·ms (fence). This was a breaching course in a single day, and there were no other activities.

While in the field training setting, in addition to demographic information in the intake form we ascertained self-report data regarding “head injury” history through the items: “Head injury, concussion or loss of consciousness,” and “your most significant head injury.” The second instance also solicits injury cause, and resulting effects on consciousness, memory, mood, sleep, all ascertained in free response narrative format. SMs approximate self-reported number of career blast exposure events prior to engagement in training were also ascertained. All participants met military physical requirement standards and were fit for duty. Finally, blood samples were collected serially at pre-blast (morning 7:30 AM to 9:00 AM), post-blast (afternoon 4:30 PM to 5:30 PM) on the training day, and at follow-up blast the next day (morning 7:30 AM to 9:00 AM) and stored in −80°C for downstream experiments. On average the post blast time point corresponds to 1 h, and follow-up to 16 h following exposures to blast during training.

***Biomarker Assays***: Details on blood collection, RNA isolation, and RNA sequencing for samples and data used in the present study have been provided previously (48). Similarly, quantification of the Aβ40 and Aβ42 levels for the data presented have been previously described (49) as digital immunoassays performed using the Simoa HD-1 (Quanterix Corporation, Lexington, MA).

***Data and statistical analyses***: All statistical and data analyses were performed using R 4.3.0 (50).

***Analysis of RNA sequencing Data***: We used voom function from R package limma to transform raw count data to logCPM matrix by computing observation-level weights based on estimated mean–variance relation. We first examined the difference in gene expression at baseline pre-blast exposure by using a linear model to compare SMs with mTBI vs. without mTBI history. Second, we examined the effect of prior mTBI history on gene expression response after blast using a design matrix that consisted of intercept, timepoint (pre-blast vs. post-blast or follow-up blast), its interaction with TBI, and subject ID. Lowly expressed genes [average logCPM ≤ 1 across all timepoints] were filtered out. The logCPM matrix was then used in an interaction model with the design matrix described above using lmFit function from R package limma. Genes with significant interaction term after adjusting for multiple testing by applying Benjamini-Hochberg adjustment were reported.

***Analysis of CNS-related biomarkers***: Biomarkers with >20% lowly expressed analytes below detectable range across all samples and timepoints were excluded, consequently resulting in exclusion of Tau. The remaining biomarkers, i.e., Aβ40 and Aβ42 were graphed for all participants and also separately by mTBI status and time points and inspected for outliers and inconsistent values. Outliers, defined as values outside 1.5 times the interquartile range above the 3^rd^ and below the 1^st^ quartiles, were winsorized - censored to the nearest non-outlier value to avoid deleting several observations from the analysis (51). For these biomarkers we: (1) assessed prior to engagement in tactical training at the pre-blast timepoint differences in biomarker levels between SMs with vs. without history of mTBI using linear models; (2) assessed the impact of blast on biomarker levels across all participants at post and follow-up blast exposure time points using linear mixed-effects models with timepoint as main effect, and subject ID as random intercept; and (3) assessed whether the impact of blast on biomarker levels at post and follow-up blast exposure timepoints differed by prior history of mTBI using linear mixed-effects models with timepoint and mTBI status both as main effects and interaction, and participant ID as random intercept. Age was adjusted in all the models as a covariate. Bonferroni corrected *p*-values were reported.

***Gene set enrichment analysis***: Genes identified with significant time by mTBI status interactions from the interaction models for pre vs. post and pre vs. follow-up separately were included in gene enrichment analyses using enrichr (52). We focused specifically on the Gene Ontology: Biological Process Database (GO:BP), used R package enrichplot package (52) for data visualization.

## Results

Serially collected biospecimen data allowed for tracking potential transcriptional regulatory changes associated with operational blast exposure from military trainees participating in an explosive breaching course (see Table 1 for participant demographic information). Participants (*n* = 30) with available blood specimen’s assayed in the present study, comprised of male SMs with average age 30.2 ± 7.4 years with 83.3% serving in the military occupational specialty (MOS) of Combat Engineer (MOS 12B). Also, prior to engagement in tactical training self-reported total number of blast exposures was recorded ranging from 2 to 60,790, as well as mTBI with 60% of participants reporting a prior history of mTBI (Table 1). No association between history of mTBI and education or MOS were observed (Table 1). SMs with mTBI vs. without mTBI history did not differ significantly in age but did differ significantly by years of military service (Table 1), showing that those with prior history of mTBI had longer duration of service in the military. There was also no significant difference in self-reported total number of blast exposures found between SMs with prior mTBI vs. without mTBI history (*p* = 0.0.2318, *t* = 1.24).

**Table 1:** Demographic and military service characteristics for participants by prior mTBI status.

	mTBI	No mTBI	Test	*p*-value
N	18	12		
Age (Years), Mean ± SD	31.61 ± 6.84	27.58 ± 4.48	*t* = 1.948	*p* = 0.061
Duration of Service (Years), Mean ± SD	10.70 ± 4.36	6.33 ± 3.68	*t* = 2.920	*p* = 0.007
Education, N (%)				
College	14 (78%)	10 (83%)	Chi-sq = 0.138	*p* = 0.709
High school	4 (22%)	2 (17%)		
MOS, N (%)				
12B/Combat Engineer	14 (78%)	11 (92%)	Chi-sq = 1	*p* = 0.317
Others*	4 (22%)	1 (8%)		
Total blast				
NA	1	1	*t* = 1.243	*p* = 0.2318
1–1,000	15	11		
1,001–10,000	0	0		
10,001–30,000	1	0		
30,000+	1	0		

*Military occupational specialty (MOS) others include 12H-Construction Engineer Supervisor, 19D-Calvary Scout, 11B-Infantryman and 31B-Military Police.

**Figure 1:**
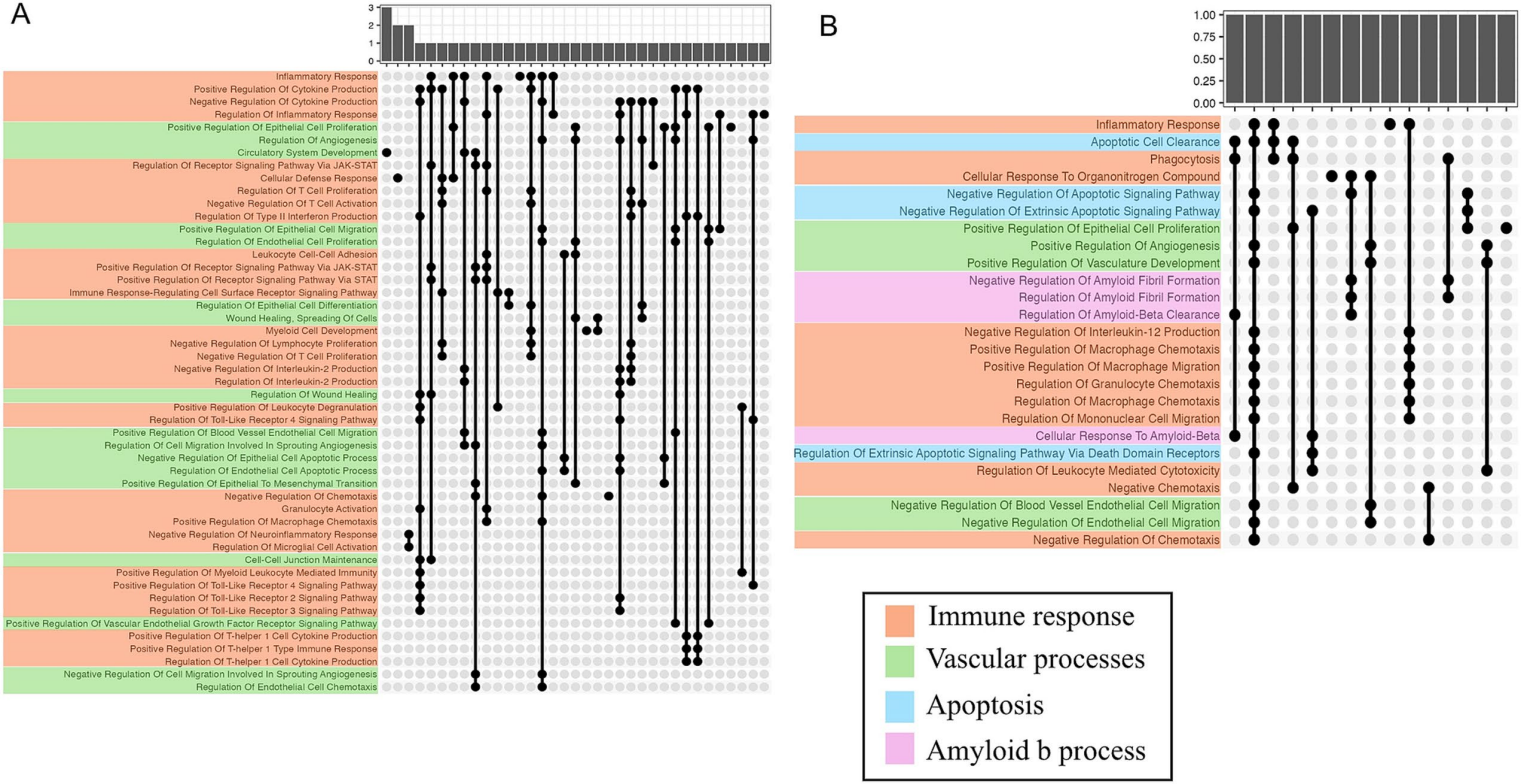
Upset plots of selected GO:BP pathways enriched with genes identified from the pre-blast vs. post-blast and pre-blast vs. follow-up-blast timepoint comparisons by mTBI status interaction model. Results are depicted with different colors by 4 functional groups: **(A)** Pre vs. post analysis identified 32 pathways involved in immune response (orange) and 17 vascular processes (green). **(B)** Pre-blast vs. follow-up-blast analysis identified 12 pathways involved in immune response (orange), 5 in vascular processes (green), 4 in Aβ regulation (pink), and 4 in apoptosis (blue). Bar plots on top show the number of gene(s) unique to or shared across pathways. Dot and line plots indicate pathways with shared gene(s), shown with the bar plots above.

**Figure 2:**
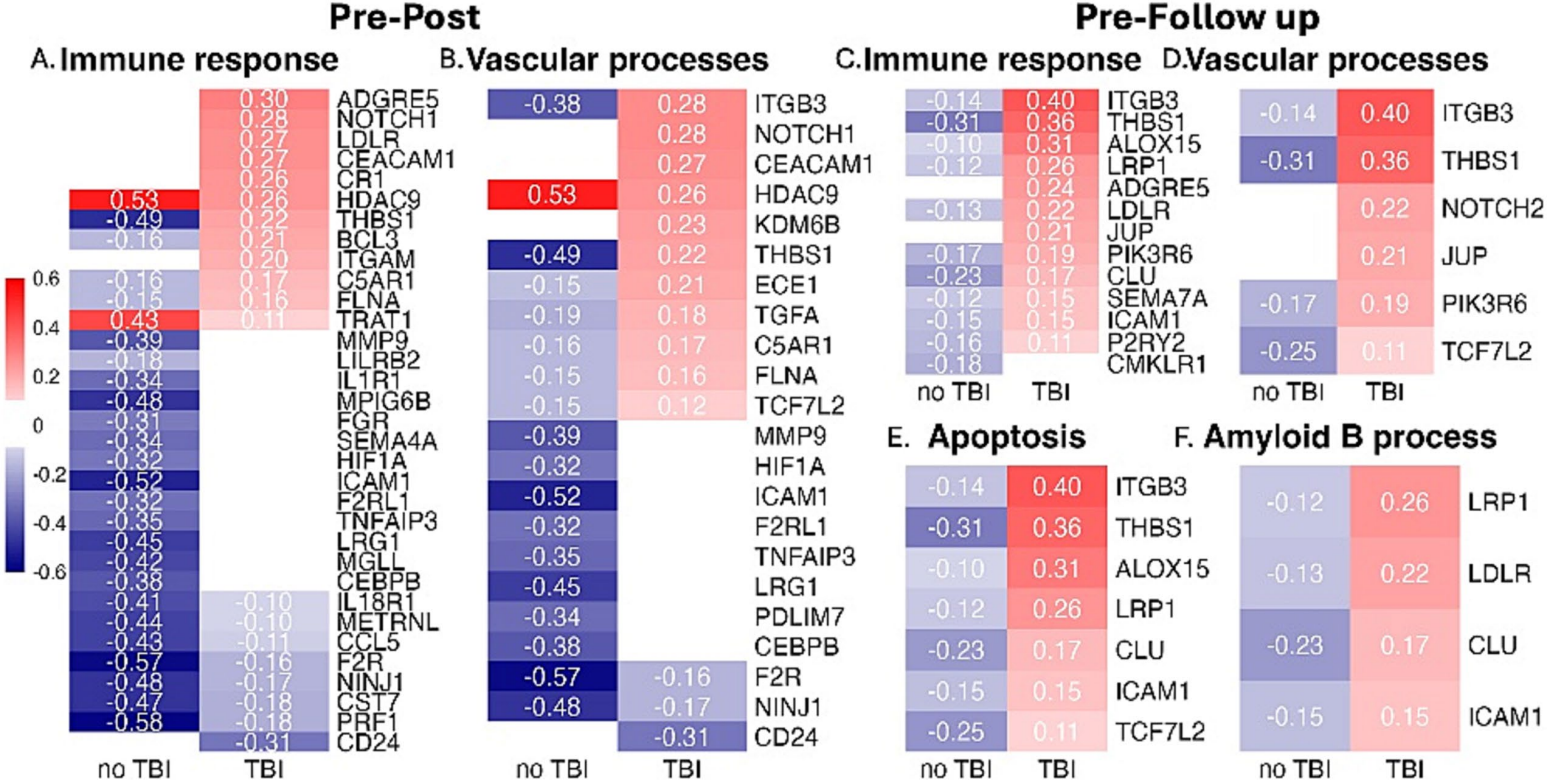
Heatmaps depicting differential gene expression among non-TBI and TBI participants for comparison of the timepoints: pre-blast vs. post-blast with enrichment of genes involved in **(A)** immune and **(B)** vascular processes, and pre-blast vs follow-up-blast with enrichment of genes involved in **(C)** immune, **(D)** vascular, **(E)** apoptosis, and **(F)** Amyloid Beta processes. Data are shown as log2 Fold change in gene expression across all comparisons.

**Figure 3:**
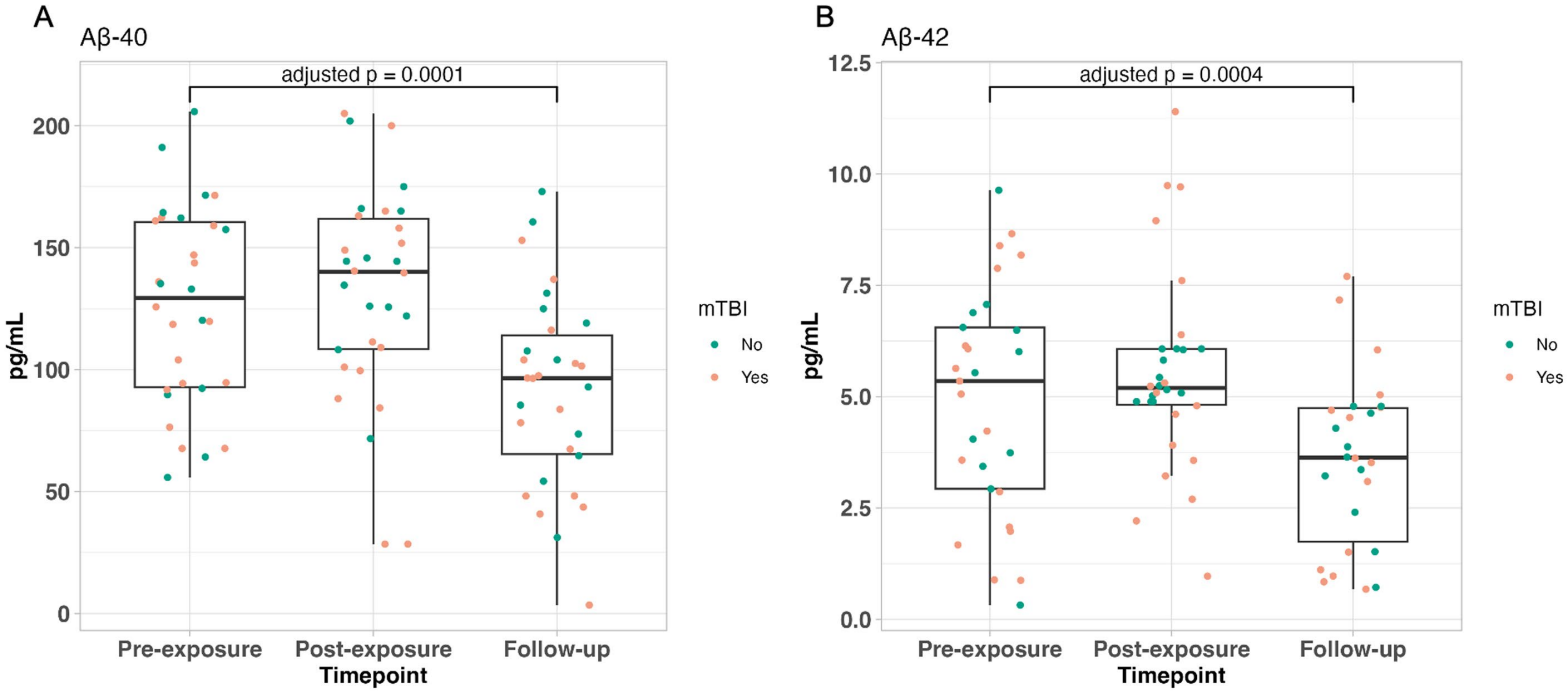
Boxplots with overlaying jitter plots show the Aβ proteins’ levels across the three timepoints. **(A)** Aβ40 levels and **(B)** Aβ42 levels across the three timepoints with overlaying jitter colored by subject’s mTBI history (green: no mTBI, orange: mTBI). *p*-values reported came from the linear mixed effect model which assessed the changes of proteins’ level between follow-up-blast and pre-blast exposure.

Comparison of gene transcript expression between SMs with or without mTBI at the pre-blast timepoint prior to engaging in tactical training only identified one differentially expressed gene (i.e., *ACCSL*). Next, to determine how prior exposure (i.e., lifetime history of mTBI) impacts differential gene expression and CNS-related biomarker responses, we used statistical models that examined the interaction of blast exposure during training and mTBI history. Among SMs with self-reported history of mTBI when compared to those without, gene expression analyses identified 130 at post timepoint and 53 genes at follow-up timepoint with significant interactions (*p* < 0.05 adjusted for multiple testing; Supplementary Tables S1, S2, respectively). Gene set enrichment analyses identified 102 (for pre-blast vs. post-blast) and 59 (for pre-blast vs. follow-up blast) significant pathways (*p* < 0.05 adjusted for multiple testing using Benjamini and Hochberg procedure; Supplementary Tables S3, S4). Among the 102 pathways identified in the pre-blast vs. post-blast analysis, 32 (31%) were involved in immune response and 17 (17%) were involved in vascular processes (Figure 1). Of the 59 pathways identified in the pre-blast vs. follow-up blast analysis 12 (20%) and 5 (8%) were involved in immune response and vascular processes respectively, and 4 (7%) were represented separately for the Aβ regulation and apoptosis pathways (Figure 2). Moreover, protein expression levels of Aβ40 and Aβ42 did not differ by mTBI history at pre-blast timepoint prior to engagement in tactical training (Aβ40: b = −14.68, t_27_ = −1.01, adjusted *p* = 0.6455, and Aβ42: b = −0.57, t_26_ = −0.60, adjusted *p* = 1). Also, prior history of mTBI did not impact the responsivity of Aβ40 and Aβ42 biomarker levels after blast exposure for the post-blast compared with pre-blast timepoints (Aβ40: b = −1.99, t_56_ = −0.12, adjusted *p* = 1; Aβ42: b = 0.70, t_52_ = 0.94, adjusted *p* = 0.7045) and for the follow-up blast compared with pre-blast timepoints (Aβ40: b = −4.31, t_56_ = −0.26, adjusted *p* = 1; Aβ42: b = 0.85, t_52_ = 1.10, adjusted *p* = 0.5540). Irrespective of prior mTBI history, significant reduction in Aβ40 and Aβ42 levels were observed at the follow-up blast exposure timepoint compared with the pre-blast exposure timepoint across all SMs (Aβ40: b = −34.76, t_58_ = −4.32, adjusted *p* = 0.0001 and Aβ42: b = −1.52, t_54_ = −4.01, adjusted *p* = 0.0004). However no statistically significant change in levels of these two biomarkers was observed acutely at the post blast exposure timepoint (~1 h) compared with the pre-blast exposure timepoint (Aβ40: b = 5.64, t_58_ = 0.70, adjusted *p* = 0.9725; Aβ42: b = 0.64, t_54_ = 1.77, adjusted *p* = 0.1642; Figure 3).

## Discussion

In the present study involving SMs engaged in explosive breaching training operations, we examined alterations in CNS-related biomarkers and transcriptome profiles serially during tactical training. Although levels of CNS-related biomarkers Aβ40 and Aβ42 were significantly altered in response to blast at the follow-up blast exposure (~16 h) timepoint in tactical training operations, these changes did not differ by the SMs prior history of mTBI. However, we did identify distinct transcriptional alterations associated with blast exposure in SMs with prior history of mTBI vs. those without. Gene transcripts that showed distinct transcriptional signatures were elevated in blood brain barrier (BBB), inflammatory and immune responses with those SMs endorsing prior mTBI history showing greater representation of these genes with heightened expression. Specifically, significant alterations in expression of genes involved in: (1) vascular processes (i.e., regulation of epithelial cell proliferation, epithelial cell differentiation, cell migration in sprouting angiogenesis, positive regulation of vascular endothelial growth factor receptor signaling, blood vessel endothelial cell migration, angiogenesis and epithelial cell proliferation); and (2) inflammatory processes (i.e., Toll-like receptor 2, 3 and 4 signaling, myeloid cell development, microglial activation, phagocytosis, macrophage chemotaxis, migration, granulocyte chemotaxis and immune response) were identified after exposures to blast. These findings point to the significance of inter-individual differences in biomarker response that capture pathophysiological mechanisms and processes associated with blast exposure, especially among individuals with greater vulnerability due to prior injuries, specifically mTBI.

Alterations of genes involved in vascular processes are consistent with findings related to BBB impairment from exposures to blast. In particular, blast shockwaves can contribute to rapid physical displacement of the blood through blood vessels from the high-pressure body cavity to the low-pressure cranial cavity, which may result in damage to the brain microvessels and the BBB (53). The BBB is a multicellular vascular structure which acts as a diffusion barrier to prevent the inflow of most compounds from blood to brain, maintaining brain homeostasis [for review, see (54–56)]. Evidence from animal models suggest that both of these mechanisms can contribute to dysfunction or disruption of the BBB and is associated with the emergence of blast-related TBI pathology (57–60). Data from both *in vitro* and *in vivo* experimental models have shown a causative relationship between exposure to blast shock waves and BBB disruption, demonstrating BBB disruption from exposure to a range of controlled blast conditions, thus providing evidence that immediate disruption of BBB can occur as a consequence of exposure to blast shockwaves (59, 61, 62). Studies in experimental models of blast using rodents exposed to low intensity blast have shown BBB disruption and led to cerebrovascular inflammation measured longitudinally 1, 6, 24 and 48 h post-blast (59). Others have found increased levels of the proinflammatory cytokines interferon gamma (IFNγ) and monocyte chemoattractant protein-1 (MCP-1) at 4 h and at 24 h post-blast with concomitant behavioral and pathological abnormalities in rodents, showing memory impairment in a novel object recognition test and increased density of Iba-1 + activated microglia in brain regions at 2 weeks post-exposure (61). Growing evidence shows that inflammation is elevated centrally and peripherally and persists after exposure to blast and possibly worsening over time. This change is mediated, in part, by microglia.

Microglia, which represent 10–13% of the cells in the CNS (63), and are innate immune cells that respond rapidly following immune challenge or injury. Microglia have numerous ramified processes which rapidly respond to local chemotactic signals (64) and constantly survey the local microenvironment (65). Microglia are derived from myeloid progenitors within the yolk sac during early embryonic development with minimal turnover over lifetime (66, 67), making them particularly susceptible to CNS injury. Microglia are functionally similar to tissue macrophages (e.g., TLR-mediated activation, scavenging, antigen presentation, clearance of misfolded proteins), which indicate a major role in immune surveillance of the brain (68–71). After acute CNS injury, microglial activation serves an important immune alert system, with the production of cytokines and chemokines (72), which are responsible for phagocytosis, scavenging of debris, angiogenesis, and wound healing. Additionally, microglia respond to damaged cells, other activated glia, and peripherally derived stimuli following BBB disruption. In response to acute injury, some “primed” microglia cells continue to maintain a heightened inflammatory state (73, 74). This is important because primed microglia have a lower threshold for becoming hyper-activated (75) in response to a secondary challenge. Microglial priming potentially plays a critical role in repeated mTBI, where the initial injury is the priming event, and subsequent injuries result in exaggerated inflammatory responses.

In line with these prior findings, in the present study we observe among SMs with mTBI dysregulation of genes and genetic pathways involved in inflammatory and vascular processes after exposures to blast. Of note, in comparison to those with no mTBI history who engaged in breacher training, in SMs with mTBI we found elevated expression of genes *THBS1*, *MMP9*, and *PIK3R6.* The *THBS1* gene encodes Thrombospondin-1 (TSP-1), a multifunctional extracellular matrix glycoprotein involved in inflammatory and angiogenic processes, and was elevated at both the post and follow-up blast exposure timepoints in SMs with mTBI history. TSP-1 (a potent anti-angiogenic agent) (76) has shown to be elevated in the brains of rodents following TBI and blood plasma from patients with TBI (77). TSP-1 levels were shown to be associated with TBI outcome and severity, delineating patients with mild, moderate, and severe TBI from healthy controls as well as worse outcomes (e.g., Glasgow Coma Scale score, cerebral hernia presence, blood glucose levels) in the 3-months following TBI (77), underscoring the potential utility of TSP-1 as a diagnostic and prognostic biomarker of brain injury. At the post-blast exposure timepoint, SMs with prior history of mTBI exhibited significant increases in expression of the gene *MMP9*, a matrix metalloproteinase. Activation of matrix metalloproteinases have been associated with neuroinflammation, vascular dysregulation, BBB breakdown, white matter damage, neuronal death, and brain edema (78). In TBI animal models, increased expression of MMP-9 has been reported with concomitant disruption in the BBB (79) and in clinical studies increased expression of MMP-9 were reported in CSF of mTBI patients compared to controls within 72 h following injury, as well as in SMs engaged in heavy weapons training with repetitive exposures to blast both acutely and chronically following blast exposure (80, 81). Furthermore, the gene *PIK3R6* encodes a subunit of the Phosphoinositide 3-kinase (PI3K) gamma complex, which was also upregulated in SMs with mTBI at the follow-up blast exposure timepoint. PI3K is involved in the activation of inflammatory responses in microglial cells, in which the upregulation of PI3K pathway has been involved in TBI and neurodegenerative disorders (82). In an rodent study of TBI, inhibition of the PI3K/akt pathway contributed to reductions in neuronal autophagy and white matter injury and corresponding improvement in functional recovery, including improvement in spatial memory and sensory coordination (83). Additionally, upregulation of the PI3K gamma is associated with neuroinflammation and cognitive deficits in experimental models of Alzheimer’s disease, where inhibition of PI3K has shown to be protective, leading to symptom improvements (84).

In addition to genes involved in immune and vascular processes, genes regulating Aβ expression were also found to be significantly modulated at the follow-up blast exposure timepoint in SMs during tactical training. Aβ is derived from the amyloid precursor protein (APP), and as a sticky protein, Aβ clumps together and forms amyloid plaques that disrupt cell function in the brain, with such plaques being a pathological hallmark of Alzheimer’s disease. In the present study, differential gene transcript expression patterns were identified in SMs with mTBI history after blast exposure (at the follow-up blast timepoint) in genes involved in regulation of amyloid fibril formation, Aβ clearance, and cellular response to Aβ production. Notably, we found that *CLU*, *LRP1* and *LDLR* genes involved in regulation of Aβ showed increased expression in SMs with prior history of mTBI at the follow-up blast exposure timepoint. The clusterin (*CLU*) gene product has been shown to facilitate reductions of Aβ levels in the CNS (85) through multiple potential mechanisms including maintaining Aβ solubility (86), preventing further aggregation of Aβ into larger oligomers or fibrils (87), regulating Aβ clearance via perivascular drainage pathways (88), and modulating CLU-Aβ phagocytosis by microglia through binding to TREM2 (89). Variants in the *CLU* gene have been associated with the risk for late-onset Alzheimer’s disease (90), with elevated CLU protein levels observed in blood and CSF of Alzheimer’s disease patients (91, 92). The *LRP1* gene encodes the Low-density lipoprotein receptor-related protein-1 (LRP1), a cell surface receptor that plays a key role in Aβ the metabolism and clearance both in the brain and in the periphery (93, 94). In particular, soluble LRP1 in plasma binds to Aβ in the periphery, preventing Aβ from entry to the brain, wherein the LRP bound Aβ accumulates in the liver and can thus be cleared systemically (94). The low-density lipoprotein receptor gene (*LDLR*) is also involved in regulation of Aβ peptide in the brain, shown through preclinical studies (95–98). These studies have shown that LDLR overexpression enhance uptake and clearance of Aβ by astrocytes, while deletion of LDLR results in decrease in Aβ uptake, suggesting the potential of *LDLR gene* as a therapeutic target for Alzheimer’s disease. Remarkably, the modulation of Aβ related pathways, and specifically the genes *CLU*, *LRP1*, and *LDLR*, after exposures to blast (at the follow-up blast timepoint ~16 h) aligns with the observed reduction of Aβ40 and Aβ42 proteins at the same timepoint. These findings suggest that exposures to blast may modulate Aβ protein expression, as also reported previously (35), that confer a compensatory molecular response to downregulate deleterious Aβ accumulation, especially among SMs with prior history of mTBI, who may be more primed to subsequent injuries.

This study has a number of limitations including a small sample size and lack of long-term longitudinal data, for both transcriptional and neurotrauma biomarkers for the breacher participants. Increased collection points several weeks or months after blast exposure training would allow for a better understanding of the dynamics and long-term effects of blast exposure on biomarker responses. The Aβ40 and Aβ42 biomarker levels in the present study are derived from serum samples, which limit the potential interpretability of clinically relevant thresholds. Although both plasma and serum have been utilized for measuring Aβ levels, comparisons of biomarker levels in the context of Alzheimer’s disease clinical research specifically have shown that some analytes, including Aβ peptides, t-tau, and multiple p-tau species present in lower levels in serum potentially attributed to loss from clot trapping (99–104). This poses a challenge for biomarker measurement in serum, in particular for SMs whose biomarker levels are close to the lower detection limit. Future investigations toward development of reference clinical thresholds across a wide age range within both civilian and military populations are warranted, as serum is more widely used in hospital systems, with more clinical tests using serum instead of plasma (105). Data on mTBI history is based on self-report and not clinician administered assessment and diagnosis. However, not all individuals who have sustained a TBI are identified at the time of initial injury. Specifically, in the case of mTBI, individuals typically do not present for medical care. Additionally, reliance on medical records is often insufficient because even when medical records are available, a large percentage of prior injuries do not receive recognition or medical attention. Therefore, self-report based on a validated screening method is currently considered the gold standard for obtaining information on lifetime history of exposure to TBI. Still, screening instruments vary in the extent to which their psychometrics have been established, lacking the reliability to capture all TBIs. Thus, in clinical settings, patient and family self-report play an important role in the diagnosis of TBI. Indeed, the subject’s self-report of previous head trauma is often used in both clinical practice and research as the primary approach for identifying incidence of TBI and related symptoms (106, 107). The study also lacks representation of both sexes, for the present cohort involved only male participants, thus, findings may not be generalizable to female SMs.

In conclusion, findings from this study underscore the importance of multimodal biomarker investigations to identify biomarkers that capture the multi-faceted pathophysiological pathways of blast related neurotrauma for discovery of novel targets that can improve characterization of injury profiles leading to personalized treatment strategies.

## Data Availability

The datasets presented in this article are not readily available because such information as the site location, data collection date, date of birth, and gender, may reasonably allow for the identification of participants in the study. Requests to access the deidentified datasets (not including these variables) should be directed to the corresponding author, on the condition that institutional and ethical requirements for sharing the data are met.

## Author disclaimer

The views expressed in this material are those of the authors, and do not reflect the official policy or position of the U.S. Government or the Department of Veterans Affairs. Material has been reviewed by WRAIR. There is no objection to its presentation and/or publication. The opinions or assertions contained herein are the private views of the authors, and are not to be construed as official, or as reflecting true views of the Department of the Army or the Department of Defense. The investigators have adhered to the policies for protection of human subjects as prescribed in AR 70-25.
